# Considerations for the Optimization of Induced White Matter Injury Preclinical Models

**DOI:** 10.3389/fneur.2015.00172

**Published:** 2015-08-12

**Authors:** Abdullah Shafique Ahmad, Irawan Satriotomo, Jawad Fazal, Stephen E. Nadeau, Sylvain Doré

**Affiliations:** ^1^Department of Anesthesiology, Center for Translational Research in Neurodegenerative Disease, University of Florida, Gainesville, FL, USA; ^2^Research Service, Brain Rehabilitation Research Center, Malcom Randall Veterans Affairs Medical Center, Gainesville, FL, USA; ^3^Department of Neurology, University of Florida, Gainesville, FL, USA; ^4^Department of Neuroscience, University of Florida, Gainesville, FL, USA; ^5^Department of Neurology, University of Florida, Gainesville, FL, USA; ^6^Department of Pharmaceutics, University of Florida, Gainesville, FL, USA; ^7^Department of Psychology, University of Florida, Gainesville, FL, USA; ^8^Department of Psychiatry, University of Florida, Gainesville, FL, USA

**Keywords:** corpus callosum, lysophosphatidylcholine, mouse, NOS inhibitor, posterior limb internal capsule, stroke, vasoconstriction

## Abstract

White matter (WM) injury in relation to acute neurologic conditions, especially stroke, has remained obscure until recently. Current advances in imaging technologies in the field of stroke have confirmed that WM injury plays an important role in the prognosis of stroke and suggest that WM protection is essential for functional recovery and post-stroke rehabilitation. However, due to the lack of a reproducible animal model of WM injury, the pathophysiology and mechanisms of this injury are not well studied. Moreover, producing selective WM injury in animals, especially in rodents, has proven to be challenging. Problems associated with inducing selective WM ischemic injury in the rodent derive from differences in the architecture of the brain, most particularly, the ratio of WM to gray matter in rodents compared to humans, the agents used to induce the injury, and the location of the injury. Aging, gender differences, and comorbidities further add to this complexity. This review provides a brief account of the techniques commonly used to induce general WM injury in animal models (stroke and non-stroke related) and highlights relevance, optimization issues, and translational potentials associated with this particular form of injury.

## Introduction

The human brain comprises both gray matter and white matter (WM), with the latter constituting roughly 60% of the total volume. Gray matter consists of neuronal cell bodies, their dendrites and axons, glial cells, and blood vessels (1). On the other hand, WM consists of myelinated and unmyelinated axons that connect various gray matter areas of the brain and support communication between neurons, as well as convey information among the network of efferent and afferent axonal fibers. Disruption of these conduction pathways may cause motor and sensory dysfunction, neurobehavioral syndromes, and cognitive impairment (2–4). In clinical settings, WM injury can occur at any time in the life span, such as with the development of periventricular leukomalacia due to hypoxic ischemic injury in infants, cardiac arrest and stroke in adults, and vascular dementia in the elderly (5–8). WM injury is the major cause of paresis in all types of stroke (9). Most obviously this is true for lacunar infarcts, which comprise about 25% of all strokes, and for lower extremity paresis in large vessel distribution strokes (except in the rare circumstance that the anterior cerebral artery territory is involved). However, because anterior circulation large vessel strokes are almost always due to clots embolizing or propagating to the carotid T-junction or the proximal middle cerebral artery, and because infarcts in both locations cause ischemia in the posterior periventricular WM, through which corticospinal and corticobulbar pathways pass, ischemic WM injury also accounts for most upper extremity paresis in large vessel distribution strokes. Furthermore, the site of periventricular WM lesions that cause paresis is also the site of crossing callosal fibers. Damage to these may contribute to apraxia after left brain stroke and may interfere with language recovery after stroke.

Thus, the extent to which WM injuries contribute to neurological impairment after stroke and the frequency with which WM damage contributes to other neurologic disorders highlights the need for therapeutic intervention strategies aimed at ameliorating WM damage or promoting WM recovery, as well as the need to dissect the molecular mechanisms involved in the pathophysiology of this injury. This review focuses essentially on techniques reported to induce WM injury. For other topics such as WM injury induced by traumatic brain injury, the pathophysiology of WM injury, WM hypersensitivity, and genetics variants leading to stroke and WM injury, which are beyond the scope of this paper, see reviews (10–17) and original research articles (18–20).

## Broad Classifications of Animal Models with WM Injuries

Attempts have recently been made to develop animal models with WM injury; however, all of these WM injury models have certain limitations. A majority of experimental studies have been designed using rodents, despite their having only about 14% of the relative WM volume of humans (1). Although disease models using primates or other higher order mammals are more readily translated to the human brain, small rodents are privileged for preclinical testing because they are cost effective and easy to acquire, handle, and monitor from a physiological standpoint. Furthermore, murine models provide researchers more flexibility in characterizing disease mechanisms because of the availability of genetically modified (transgenic or knockout) animals. The variety of methods used to induce WM injury in rodents reflects not only the complexity of the disease, but also the need for different paradigms to identify mechanisms that cause WM damage. Such considerations provide the foundation for the development of preclinical novel therapeutic approaches. In general, WM lesion models can be classified into two main categories, as discussed below.

### Focal WM injury

**Figure 1:**
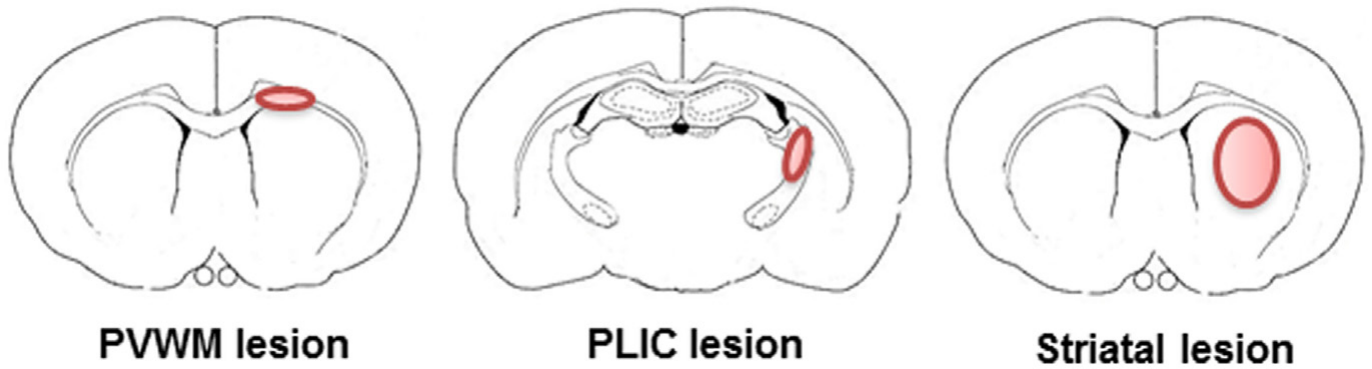
**Representative illustrations of targets to induce focal white matter injury in rodents**. In rodents, only a small percentage of the brain is WM and WM is mostly located in the periventricular white matter, posterior limb internal capsule, and striatum. Therefore, all of the rodent models of WM injury employ one or more of these areas.

Focal WM injuries are produced at a specific locus in a WM region of the brain. Periventricular WM (PVWM) and posterior limb internal capsule (PLIC) are the most commonly targeted areas to produce injury in WM injury models (Figure 1). PVWM injuries are produced by targeting the corpus callosum (CC). The PLIC, which contains corticospinal tracts from the motor cortex, can be targeted to induce motor deficits that are important in determining the efficacy of a therapeutic approach when dealing with functional outcomes (see Table 1).

**Table 1:** **Preclinical models of white matter brain injury**.

Focal white matter injury	Animal	Lesion location	Morphological changes	Functional deficits, reference
**ENDOTHELIN 1 (ET-1)**				
	Mouse	CC/PVWM	Gliosis, axonal degeneration, myelin loss	No report (21)
	Mouse	Subcortical WM, striatum	None	No report (22)
	Rat	CC, striatum	Gliosis, demyelination	No report (23)
	Rat	PLIC	Demyelination	Yes (24)
	Rat	PLIC	Lesion	Yes (25)
	Rat	PLIC	Axonal degeneration, myelin loss	Yes (26)
**AchA OCCLUSION**				
	Rat	IC, lateral and medial hypothalamus	Infarct	Yes (27)
	Pig	IC	Infarct	Yes (28)
**PHOSPHATIDYLCHOLINE**				
*LPC*	Rat	CC/PVWM	Demyelination	No report (29, 30)
	Mouse	CC/PVWM	Demyelination	No report (31)
**NOS INHIBITOR**				
*L-NAME* + ET1	Mouse	Subcortical WM, striatum	Infarct	Yes (22)
*L-NIO*	Mouse	Subcortical WM, CC	Infarct, demyelination	No report (32)
**OTHER**				
*Myelin debris*	Rat	CC/PVWM	Gliosis, demyelination	No report (33)

**Global white matter injury**	**Animal**	**Lesion location**	**Morphological changes**	**Functional deficits, reference**

**CHRONIC HYPOPERFUSION**				
*Bilateral CCA ligation*	Rat	CC, IC, caudate-putamen	Hippocampal cell death	Cognitive impairment (34)
	Mouse	Optic tract, CC, IC	Demyelination, gliosis	No report (35)
	Mouse	Hippocampus	Neuronal death	Memory loss (36)
	Gerbil	Hippocampus, Cortex	Gliosis, neuronal death	Yes (37)
	Gerbil	Whole brain	Ventricular dilation, atrophy, gliosis, demyelination, NF degeneration	No report (38, 39)
*Asymmetric CCA ligation*	Mouse	Subcortical WM	WM rarefaction, gliosis, axonal damage	Motor and cognitive deficits (40)
**HYPOXIC-ISCHEMIC INJURY**				
*Unilateral ligation* + hypoxia	Rat	Cortex, striatum	Cell death, atrophy	Yes (41)
	Rat	Hippocampus	Demyelination	Memory loss (42)
	Mouse	CC, hippocampus, Thalamus	Demyelination, necrosis	No report (43)
*Hypoxia only*	Rat	CC, PVWM	NOS, gliosis, axonal degeneration	No report (7)
**LPS**				
	Rat	CC	Demyelination (delayed)	Acute motor deficits (44)
	Sheep	PVWM, cortex	Inflammation, gliosis	Cardiovascular changes (45)
	Sheep	IC, EC	Demyelination (delayed)	No report (46)

*AchA, anterior choroidal arteries; CCA, common carotid artery; CC, corpus callosum; EC, external capsule; IC, internal capsule; L-NAME, L-N^G^-nitroarginine methyl ester; L-NIO, L-N^5^-(1-iminoethyl)ornithine; LPC, lysophosphatidylcholine; NF, neurofilament; NOS, nitric oxide synthase; PLIC, posterior limb internal capsule; PVWM, paraventricular white matter; WM, white matter*.

### Global WM injury

Global WM injuries are reflected by diffuse axonal injury occurring in the entire brain as a result of hypoxic/ischemic brain injury, heightened inflammatory states resulting from autoimmune diseases or infection, and/or exposure to demyelinating agents. It is well recognized that the perinatal WM is susceptible to hypoxic/ischemic injury, especially in the preterm period (47). A well-established model of perinatal hypoxic/ischemic injury is unilateral ligation of one common carotid artery (CCA) in combination with hypoxia in the neonatal rodent. It will result in a decrease in cerebral blood flow (CBF) in the middle cerebral artery territory, which corresponds to clinical birth asphyxia (48). Diffused WM injury may also occur in global hypoperfusion, which commonly occurs in cardiac arrest patients. In this context, Shibata and colleagues developed an adult rodent model of chronic cerebral hypoperfusion that provided further insight into mechanisms of WM injury pathogenesis (35).

## Ischemic Stroke-Related Focal WM Injury Models

Determining the damage to specific regions of motor pathways can help predict potential functional outcomes. An animal model of a focal WM infarct has been developed using stereotaxic injections of selective drugs in rodents. Stereotactic delivery of selective vasoconstrictors or demyelinating agents induces the local degeneration of WM in a distinct region of the brain by ischemia or the loss of myelin. Subcortical WM, such as the PVWM and the PLIC, contains descending axonal fibers originating in the motor cortex and ascending fibers conveying sensory information to the somatosensory cortex. Therefore, the loss of these connections, as observed in stroke in general and in WM injury in particular, results in a significant clinical phenotype reflected by substantial sensorimotor deficits. Considering the importance of these networks and connections, it is important to develop a WM injury model by targeting PVWM or PLIC.

### Target brain regions to induced focal WM injury

#### Paraventricular White Matter

PVWM refers to the WM adjacent to the bodies of the lateral ventricles. It includes ascending and descending fibers in continuation with the internal capsule (the corona radiata), callosal fibers, and, more laterally, long anterior–posterior fasciculi.

Periventricular leukomalacia in infants is caused by a decrease of blood or oxygen flow during the perinatal period and is characterized by necrosis, astrogliosis, microglial infiltration, and depletion of premyelinating oligodendrocytes in the PVWM (49, 50). Periventricular leukomalacia most often occurs in premature infants. It is associated with neurodevelopmental impairments such as cerebral palsy, with or without mental retardation, learning disability, or epilepsy (48, 51). White matter injuries in PVWM are associated with cognitive and emotional decline (52, 53). Executive, and to a lesser extent, episodic memory formation and declarative memory retrieval are particularly susceptible to deep WM injury because of the particular dependency of these functions on the integrity of long WM pathways (54).

In an effort to develop relevant animal models, several studies have successfully induced infarction and/or demyelination by targeting the PVWM (21, 29–31). Unfortunately, in contrast to what has been reported in the human studies, these animal lesion models have not been associated with significant functional deficits (Table 1), and further refinement is warranted.

#### Posterior Limb Internal Capsule

In humans, the PLIC contains corticospinal fibers and sensory projections from the thalamus deriving from both lemniscal and anterolateral systems. The anterior limb of the internal capsule includes frontopontine and thalamocortical projections, which modulate higher cognitive functioning (55). The PLIC is vascularized by the anterior choroidal branches of the internal carotid artery and lenticulostriate branches of the middle cerebral artery. Damage to corticospinal motor fibers in the PLIC results in sensorimotor deficits in patients. In human stroke, damage to sensorimotor fibers passing through the PLIC is by far the most important contributor to hemiplegia or hemiparesis, although this damage most often occurs more rostrally, in the PVWM or the centrum semiovale (9). Paresis is characteristically worst in the arm but often involves the face and lower extremity. Dysarthria, dysphagia, and sensory symptoms may also be present in these patients (8). With stereotaxic injection of the vasoconstricting agent endothelin 1 (ET-1) in the PLIC, Frost et al. (24) and Lecrux et al. (25) induced infarction in the PLIC that was associated with functional deficits (24, 25). Thus, selective targeting of the PLIC by vasoconstricting or demyelinating agents can be used to induce a reproducible WM injury model with anatomical and functional deficits.

### Vasoconstricting agents used to induce focal WM injury

Vasoconstrictors have been reported to selectively induce focal WM injuries. Potent vasoconstrictors are potential tools that need to be fully exploited to further evaluate the mechanisms associated with ischemic WM injury and potential therapeutic treatments. The following is a list of some of the vasoconstrictors that are commonly being used to develop WM injuries in animals.

#### Endothelin 1

Endothelin 1, a 21-amino acid peptide that is produced by vascular endothelial cells, is a potent vasoconstrictor (56–58). It participates in the development of atherosclerosis, hypertension, and vasospasm following subarachnoid hemorrhage (59). ET-1 coexists with other isoforms, ET-2 and ET-3, which are synthesized by a variety of cells, including glial cells and neurons (60–63). Within the central nervous system, ET-1 is distributed widely within the brain and spinal cord (60, 64–66), but it cannot cross the blood–brain barrier (67, 68). Two classes of ET receptors, ET_A_ and ET_B_, exist in many tissues. The ET_A_ receptor has a high affinity for ET-1 and ET-2, but a low affinity for ET-3, whereas the ET_B_ receptor has equal affinity for all three isoforms (69).

At present, the cellular mechanism of ET secretion in neurons and glial cells is poorly understood because the content of ET in neurons and glia is far less than what is found in endothelial cells. ET is thought to bind to its receptors and mobilize intracellular calcium via phospholipase C activation and/or through calcium channels (70). Application of ET-1 in the subcortical WM (such as the PVWM or PLIC) will reduce blood flow, disrupt myelin, and cause axonal injury (12, 71). When ET-1 is administered via intraparenchymal injection or applied topically to cerebral blood vessels, it elicits a sustained vasoconstriction and leads to a monophasic reduction in microvascular blood flow (72–75). Furthermore, the hypoxia of endothelial cells surrounding cerebral vasculature after infarction will trigger *de novo* synthesis of ET-1 and contribute further to vasospasm-induced brain ischemia (76). The application of ET-1 has been shown to effectively induce stroke in rats (75, 77, 78). However, additional precaution should be taken while considering this model because application of ET-1 topically or in brain parenchyma can lead to non-specific jury to the WM and gray matter. Indeed, when ET-1 or other vasoconstriction agents are applied in brain parenchyma, such as striatum, it affects the cerebral fibers network; however, other striatal circuitries are also affected non-selectively. A striatal-induced stroke/lesion is likely a combination of a white and gray matter lesion. The bundles of penetrating corticopetal fibers in the rodent striatum are indeed injured or destroyed in such a model. Therefore, in an effort to develop a focal WM infarction model that can mimic the lasting neurologic deficits, which is a clinical feature of WM stroke, a recent report in rodents shows that ET-1 injection into the internal capsule results in focal infarction and severe axonal and myelin loss. Such a rodent model also exhibit significant functional deficit at 1 month post-injection (26). However, when ET-1 is applied to cortical tissue in mouse brain, it has produced inconsistent results (22, 79), suggesting dissimilarities between mice and rats in response to ET-1 because of differences in expression of ET-1 receptor isoforms (80). On the other hand, a combination of ET-1 and L-N^G^-nitroarginine methyl ester [L-NAME, a nitric oxide synthase (NOS) inhibitor] increased the probability of producing an infarct in mice; however, the lesion was still relatively small and strain dependent (22).

#### Nitric Oxide Synthase Inhibitors

Nitric oxide synthase plays important roles in physiological and pathological events, including blood pressure homeostasis, neurotransmission, and immune function in the central nervous system (81). Endothelial NOS (eNOS) is found in the endothelium of cerebral vessels and also in a subset of neurons. Nitric oxide (NO) generated by eNOS is crucial for vascular function and hemostasis. By contrast, NO produced by the neuronal and inducible isoforms of NOS can be neurotoxic (82, 83). Furthermore, overproduction of NO may also have pathological consequences (84). Nevertheless, NO plays a critical role in neuroprotection against ischemic stroke through various processes, including ischemic pre- and post-conditioning (85–87). Consequently, some of these NOS inhibitors are being used to produce focal ischemic brain damage.

L-NAME is an arginine analog commonly used as a potent inhibitor of NOS. It has been shown to induce a rapid rise in blood pressure and a decrease in cardiac output (88, 89). It has been reported that a striatal injection of 100 mM L-NAME alone in the rat produces minor injury, whereas when it is co-administered with quinolinic acid (an NMDA receptor agonist), it augments striatal injury (90). Similarly, co-administration of L-NAME with ET-1 augments infarct volume. However, it has also been reported that the final outcomes following such combinational treatments are strain dependent and produce a relatively small lesion size (22). By contrast, L-NAME has also been shown to have neuroprotective effects in rodent models of cerebral ischemia. It has reduced the effect of ischemic brain injury and prevented blood–brain barrier disruption in animal models (91–93).

L-N^5^-(1-iminoethyl)ornithine (L-NIO, a selective eNOS inhibitor) is approximately five times more potent as an eNOS inhibitor than any other arginine analog, including L-NAME (94). The inhibitory effects of L-NIO are rapid in onset and irreversible, in contrast to other arginine analogs (95). Carmichael and colleagues demonstrated that microinjection of L-NIO in either the cortex or CC induced focal cortical or callosal WM stroke (32). This microinjection resulted in axonal fiber loss and myelin damage within the CC.

## Non-Ischemic Stroke-Related Focal WM Injury Models

### Ethidium bromide

Ethidium bromide (EB) has been used extensively to induce demyelination in animal models because of its cytotoxic effect on cells. Many reports have shown the effectiveness of EB-induced focal demyelination in various WM regions of the brain (96–99). The size of the injury is dependent on the volume and concentration of EB, and survival time points (100). Compared to other demyelinating agents, EB administration results in relatively delayed remyelination. Oligodendrocyte and astrocyte loss occurs within the epicenter of the lesion. Neurons and endothelial cells are less sensitive than glia, and axons remain unaffected (101–103).

### Myelin-enriched brain debris

This method was recently introduced by Clarner and colleagues (33). Myelin-enriched debris taken from the CC of 7-week-old C57BL/6 mice is homogenized with Precellys**©** zirconium oxide beads. After extraction of the protein contained within the debris using the Bradford method, 6500 μg/mL of protein in a total volume of 1 μL was stereotactically injected into the CC or the cortex of deeply anesthetized animals (33). This direct application of myelin-enriched brain debris induces profound myelin loss and inflammation.

## Demyelinating Agents Used to Induce Focal WM Injury

### Lysophosphatidylcholine

The partial hydrolysis of phosphatidylcholines generates lysophosphatidylcholine (LPC), which is also known as lysolecithin. This hydrolysis is generally the result of phospholipase A_2_ enzymatic action on phospholipids, which generates free fatty acids such as arachidonic acids and lysophospholipids such as LPC (104). LPC is believed to play an important role in atherosclerosis and inflammatory diseases by altering various functions in a number of cell types, including monocytes, macrophages, and T-cells (105). LPC is a potent demyelinating agent and has been used widely to study various aspects of demyelination and remyelination in the mature rodent central nervous system (106, 107) as well as *in vitro* organotypic cerebellar slice culture (108). LPC induces demyelination of the brain by the action of recruited macrophages and microglia, which phagocytose nearby myelin. Several reports have demonstrated that microinjection of LPC in the PVWM and WM regions of the spinal cord results in demyelination, an increase in proinflammatory genes, and the induction of gliosis (29–31, 109–111).

A significant limitation of LPC-induced injury, at least from a stroke and rehabilitation point of view, is the potential for reversibility of the injury. Lesions rapidly become repopulated with oligodendrocyte precursors, which initiate remyelination. In rodents, remyelination begins 7 to 14 days post-lesion, depending on the location of the lesion and the age of the animal. Most of the remyelination is essentially complete by 3–4 weeks (112).

## Ischemic Stroke-Related Global WM Injury

### Chronic cerebral hypoperfusion model

Neonatal WM damage in the periventricular region is often identified as periventricular leukomalacia. Factors predisposing patients to periventricular leukomalacia include immature cerebrovascular development and reduction of CBF in association with hypoxic/ischemic insults such as birth trauma, asphyxia, respiratory failure, cardiopulmonary defects, and premature birth and/or low birth weight (49). The pathological processes involved in global WM injury are still unknown. However, the intrinsic vulnerability of oligodendrocyte precursors to hypoxic/ischemic injury is considered to be central to the pathogenesis of periventricular leukomalacia. These cells are susceptible to a variety of injurious stimuli, including free radical exposure, the lack of trophic factors, and excitotoxicity resulting from cerebral hypoperfusion, as well as secondary events consequent to microglial activation and astrogliosis (49). The increased expression of proinflammatory cytokines (such as TNFα, IL-1β, and IL-6) in amniotic fluid has also been reported as an identifiable risk factor for the development of brain WM injury (113).

Since the development of the first rodent neonatal hypoxic/ischemic brain injury model, this model has been routinely used to better understand the neuropathology of stroke in neonates (51, 114, 115). The injury in neonatal rat pups at postnatal day 7 (P7) is induced through unilateral ligation of the CCA followed by creation of hypoxia (6–8% oxygen mixed with nitrogen) for a certain period of time (51, 114, 115). Brain damage, seen histologically, is generally confined to the cerebral hemisphere ipsilateral to the arterial occlusion. Depending on the duration of the hypoxia, injury may include the cerebral cortex, hippocampus, striatum, and thalamus. The duration of hypoxia determines the extent and severity of WM damage observed in subcortical and PVWM. The procedure was later adapted to the preterm equivalent rat at P2, which corresponds to the developmental stage/phenotype present in a preterm infant. At this time, highly vulnerable pre-oligodendrocytes are present in high numbers. In these circumstances, damage occurs throughout cerebral gray and WM (116) and is associated with behavioral deficits (41).

Hypoperfusion models in adult animals involving bilateral CCA stenosis have been introduced by Hattori and co-workers (37) and by Shibata and colleagues (35). Microcoils are wrapped around both CCA below the carotid bifurcation in mice to reduce the lumen diameter. This method results in a chronic reduction of CBF in the brain and induction of WM injury after 14 days without any gray matter involvement. In this model, the most severe WM injury occurs in the medial portion of the CC adjacent to the lateral ventricle and in the PVWM; moderate lesions are found in fiber bundles of the caudate-putamen and internal capsule and less severe lesions develop in the anterior commissure and the optic tracts (35). Interestingly, Yatomi et al. (117) reported that ligation of both CCA in rats decreased CBF by up to 60% with gradual improvement in flow over time. However, CBF never returned to baseline despite a survival time of 28 days. Following chronic cerebral hypoperfusion, blood–brain barrier disruption, especially in the CC, along with glial activation, may contribute to the pathogenesis of WM injury (118). A recent asymmetrical hypoperfusion model by Ihara and colleagues reports the use of a microcoil on one CCA while an ameroid constrictor was applied on the other CCA. Ameroid constrictor absorbs water and swells, resulting in the constriction of the CCA while use of a microcoil acts as vascular stenosis. The use of these two different approaches in the same mouse led to a gradual decrease in CBF and by day 28, these mice developed functional deficits and WM degeneration (40).

## Non-Ischemic Stroke-Related Global WM Injury

### Hypomyelination induced by lipopolysaccharide in the developing brain

The pathogenesis of periventricular leukomalacia appears to be multifactorial. Two important factors are hypoxic/ischemic injury and maternal-fetal infection. It has been hypothesized that maternal infection during the preterm period can lead to fetal brain injury. Clinical, epidemiological, and neuropathological data support maternal-fetal infection as an important pathogenic factor in infants with periventricular leukomalacia. Maternal infection increases proinflammatory cytokines levels in amniotic fluid and fetal blood and is associated with WM disorders such as periventricular leukomalacia (113, 119). Animal models of periventricular leukomalacia-like WM injury can be mimicked by injecting lipopolysaccharide (LPS) into the intraperitoneal cavity of pregnant rodents (120, 121) or by intravenous injection in ovine fetuses (45, 46). LPS has been reported to cross the placental barrier and induce systemic inflammation (45, 122). Maternal injection of LPS at gestational day 18 or 19 (G18 or G19) increases proinflammatory cytokines such as TNFα, IL-1α, and IL-6 and leads to generation of reactive oxygen species by inducing depletion of peroxisomes in premyelinating oligodendrocytes in the fetal brain (113, 120, 123). Apoptosis has also been reported to be a contributing mechanism of cell death in infants with WM injury (124). Both *in vitro* and *in vivo* studies have shown that LPS causes significant toxicity to premyelinating oligodendrocytes, suggesting that these cells are more vulnerable to proinflammatory cytokines and/or oxidative stress than mature cells (125, 126). Furthermore, astrogliosis and microglial activation are hallmarks of neuroinflammation and are associated with the apoptosis of premyelinating oligodendrocytes that occurs in the wake of maternal LPS exposure (44).

## Functional Deficits in the WM Injury Model

Preclinical research on stroke has predominantly focused on models that replicate the vascular events experienced by humans. However, because of the enormous anatomic differences between rodent and human brains, the neuropathological consequences of induced vascular events in rodents are substantially different from those in patients. Thus, traditional rodent models may not be optimal for the study of prognosis or neurorehabilitative intervention. More targeted approaches are needed. Because paresis in human stroke is substantially attributable to WM ischemic injury, the development of ischemic stroke models in rodents that predominantly involve WM is essential if we are to make progress in rehabilitation of paresis after stroke. If, on the other hand, the rehabilitation focus in patients is behavioral deficits wrought of gray matter injury (e.g., aphasia, apraxia, impairment in visuospatial function, hemispatial neglect, and deficits in emotional function), then the traditional middle cerebral occlusion model in rodents, which produces predominantly gray matter injury, may be optimal. Furthermore, because of the degree of “cerebralization” of function in the human brain, it is simply not possible to recapitulate the functional consequences of human hemispheric stroke in a rodent model. For example, most patients with middle cerebral artery stroke permanently lose all or nearly all upper extremity function. Rodents with the analogous vascular lesion demonstrate modest and transient impairment. The central experimental challenge in rodent models is to develop functional measures sufficiently sensitive to reveal differences between treatment and control groups weeks or months following the injury.

To study rehabilitation therapies, suitable animal models of WM injury to produce functional deficits in defined brain circuits and functional improvement following therapeutic intervention are needed. For example, in human stroke, the type and extent of behavioral deficits are dependent on different parts of the somatosensory cortex related to the lesion locus. In global WM injury, in which most of the injury will encompass many functional circuits in the brain, morphological changes as well as functional deficits will become evident (Table 1). However, in focal WM injury models, results are inconsistent and depend on the techniques, drugs/chemicals used, and the location of lesions. The application of ET-1-induced middle cerebral artery occlusion is a standard ischemic model that also induces WM damage. This method is relatively less invasive, and important parameters related to reperfusion can be easily tested (127, 128). Similarly, occlusion of the anterior choroidal arteries (without compromising the anterior or middle cerebral artery origins) exclusively produced infarction in the striatum, including the internal capsule in rats (27) and pigs (28). Acute damage to the PLIC can be a useful predictor of motor outcomes and prognosis for stroke patients (8). ET-1 has been successfully used to induce PLIC damage and produce a functional deficit (24, 25). However, injury in the CC or PVWM in animal models does not result in behavioral deficits (29–31), in contrast to patients with paraventricular leukomalcia, who do exhibit significant behavioral deficits. One of the important reasons for this discrepancy could be the substantial differences in the composition of the gray and WM in humans and rodents. The PLIC injuries identified in current studies are more likely to demonstrate functional deficits than the PVWM injuries; therefore, PLIC injuries represent a viable model for measuring motor deficits.

## Mechanisms of WM Injury

Innovative histological techniques have provided the foundation for current cellular and molecular studies on the role of proinflammatory cytokines, gliosis, reactive oxygen species, glutamate toxicity-induced oligodendroglial cell death, and demyelination in WM. Since the early 1970s, Leviton and Gilles have systematically studied PVWM injury and subsequent neurological disability (e.g., cerebral palsy and cognitive deficits) in premature infants (5, 129–132). The spectrum of chronic PVWM injury includes focal cystic necrotic lesions and diffuse demyelination. The demyelination process will then provoke the proliferation and migration of microglia into the lesion, and astrocytes around the lesion (133, 134).

**Figure 2:**
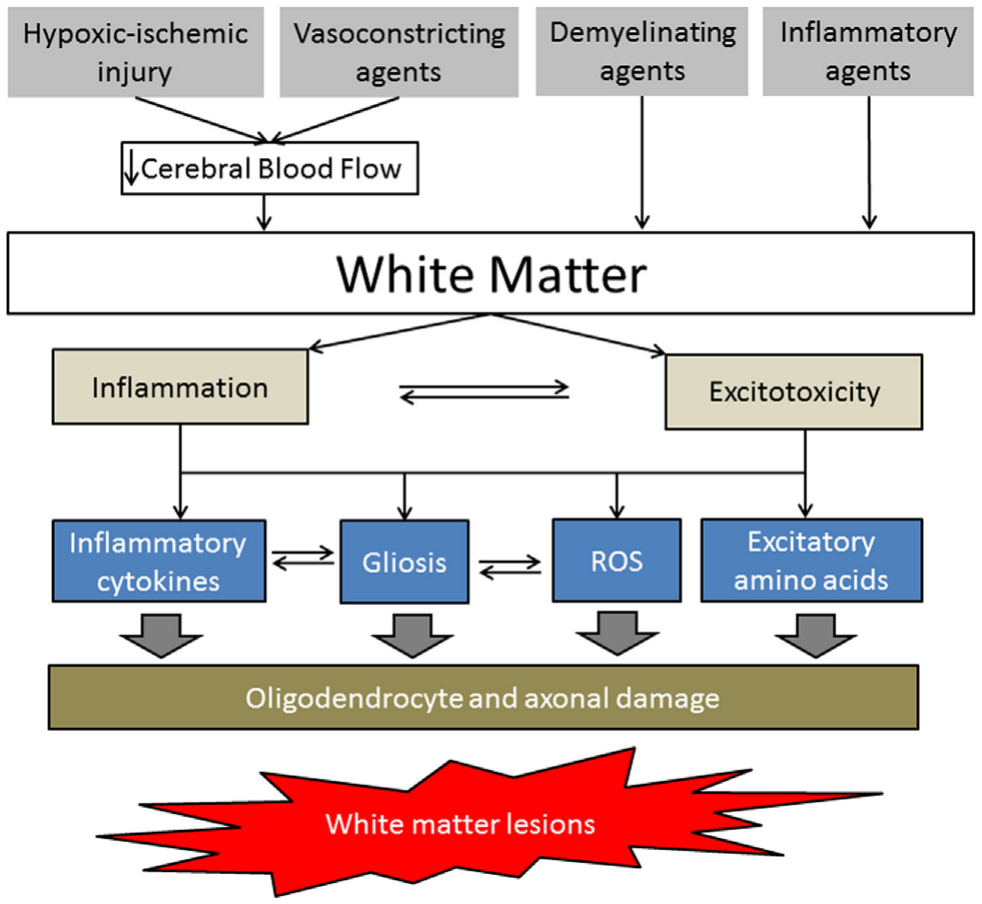
**Simplified schematic of white matter injury pathogenesis**. The hypoxic-ischemic injury and vasoconstricting agents decrease cerebral blood flow, resulting in energy depletion and blood–brain barrier disruption in the WM. Demyelinating and inflammatory agents directly affect WM by increasing proinflammatory cytokines, gliosis, reactive oxygen species, and/or excitatory amino acids, leading to oligodendrocyte precursor and axonal damage.

The pathways (intrinsic and extrinsic) that regulate the response of the oligodendrocyte lineage during initial and ensuing phases of injury (including myelination failure) remain unclear. Further neuropathological studies are needed to provide guidance for developing animal models of WM injury. There is no definitive explanation of the mechanism of the WM damage caused by injury to oligodendrocytes produced by such acute neural insults, but based on some preliminary studies, it is believed that at least four major factors can contribute: (1) hypoxic/ischemic injury, (2) vasoconstriction (that may be induced by agents such as ET1, NOS inhibitors, etc.), (3) direct demyelination (that may be induced by agents such as LPC, EB, etc.), and (4) inflammatory processes (maternal-fetal infection induced by agents such as LPS) (Figure 2).

Hypoxic/ischemic conditions and vasoconstrictor agents reduce CBF to the target area of the brain and induce WM injury. WM of the mammalian central nervous system suffers irreversible injury when subjected to anoxia/ischemia (19, 135, 136). Furthermore, WM in older adult mammals is more susceptible to ischemic injury than that in younger adults (137). Ischemia reduces adenosine triphosphate (ATP) and precipitates glutamate release due to reverse Na-dependent transport (136, 138), resulting in the overactivation of AMPA/Kainate receptors. This excitotoxic condition then causes the accumulation of Ca^2+^ in cytoplasm and triggers oligodendrocyte death and axonal disruption (7, 135, 136, 139, 140).

The role of inflammation and excitotoxicity following WM injury has been suggested in numerous reports. Evidence suggests that several proinflammatory cytokines, including IFNγ, TNFα, IL-1β, IL-2, and IL-6, are elevated in periventricular leukomalacia (113, 141–143) and may play pivotal roles in perinatal WM injury (49, 144, 145). Microglial activation and astrogliosis have also been reported in prenatal periventricular leukomalacia (71, 146, 147) and in an animal model of this disorder (114). Moreover, it has also been shown that glial cells express glutamate receptors and are thus susceptible to excitotoxicity following hypoxic/ischemic insult or treatment with a demyelinating agent (140, 148, 149).

There is ample evidence suggesting that oxidative stress correlates with the inflammatory response to ischemia-induced WM injury. Cerebral WM is susceptible to oxidative stress that particularly targets the immature stages of oligodendrocyte development. In addition, following hypoxic/ischemic injury, oxygen-free radicals contribute to chronic cerebral hypoperfusion-induced WM injuries via direct cytotoxic damage involving lipid peroxidation and by induction of inflammatory processes (14, 16, 140, 149–151).

## Various Techniques Employed to Investigate WM Injury Outcomes in WM Preclinical Models

Because preclinical and clinical investigations have pointed toward the significant role of WM in various physiologic and pathophysiologic conditions, these studies have primarily utilized novel imaging and neuropathologic tools to better understand the mechanisms of WM injury. In the following sub-sections, some of these studies have been summarized to familiarize the reader with these techniques; however, for greater in-depth understanding of these techniques, please refer to some of these articles (12, 14, 151–154).

### Imaging

Imaging techniques have improved substantially and have paved the way for the analysis of WM functions. Diffusion tensor imaging (DTI) has been used to determine WM degeneration in a mouse model of hypoperfusion (155). Similarly, in a model of neonatal hypoxic/ischemic brain injury, WM microstructure integrity was assessed by MRI (156). Traumatic brain injury also leads to microstructural disruption in WM and these changes can be assessed by DTI (157, 158). Delayed WM injury can lead to significant functional deficit; therefore, early detection of such impairments is highly recommended. In a model of hypoxic/ischemic brain damage, delayed WM injury is detected by longitudinal diffusion tensor and manganese-enhanced MRI (154). In a mouse model of WM injury, ET-1 was injected in the subcortical WM and the axonal damage was studied by performing MRI (21). The use of such imaging techniques has improved the diagnostics of WM injury and provided the greater details of structural changes post-ischemic and other neurologic conditions.

### Neuropathology

In preclinical models, several neuropathologic techniques have been used to test various parameters to determine WM injury. In a model of TBI, axonal disruption in WM was assessed by transmission electron microscopy (TEM) or immunostaining with various specific markers (e.g., NF200), or by performing Luxul Fast Blue staining (158). In a recently described model of hypoperfusion leading to WM injury and dementia, the severity of the WM lesions was determined by performing immunohistochemistry for the markers of astrocytes (anti-GFAP antibody), microglia (anti-Iba1 antibody), oligodendrocyte (anti-GST-π antibody), and damaged axons (anti-neurofilament H non-phosphorylated (SMI32) antibody) (40). The damage in WM resulted from hypoperfusion leading to vascular cognitive impairment and was quantified by performing staining for myelin basic protein (anti-MBP) (159). Several other immunohistochemical markers have been targeted to understand some of the mechanisms associated with WM injury (160–163). These well-established immunohistochemical protocols provide greater detail into the role of WM in acute neurologic conditions and help in better understanding the mechanisms associated with WM injuries.

## Optimization of Induced WM Injury

As discussed above, several models of WM injury induced by vasoconstricting agents, demyelinating agents, or vascular manipulations have been reported. The extent of injury is dependent on many factors, including the drugs or agents administered, brain areas targeted, gender, age, and survival time point post-injection. Consequently, most of these studies report anatomical outcomes and only a few studies correlate anatomical outcomes with functional changes (Table 1). Use of agents such as ET-1, LPC, or L-NIO, in addition to other compounds/strategies and their related injection sites, provide a plethora of information that has improved our understanding toward the pathophysiology of WM injury during stroke. However, WM injury models that can be truly referred as a WM injury model of ischemic stroke need further refining. Therefore, some considerations, as discussed below, should be taken into account for optimizing an induced WM injury model relevant with stroke.

First, it is important to choose a brain region that is mostly affected during ischemic stroke. One such area is PLIC or IC. This brain region is an important area to control motor functions and is substantially affected soon after the onset of stroke (164–167). Therefore, targeting this WM brain area will most likely provide a better and consistent WM injury model. The second aspect to consider is to have a better choice of WM injury-inducing chemicals (such as ET-1, L-NIO, or LPC). The advantage with ET-1 and L-NIO, in terms of stroke research, is that these vasoconstricting agents result in irreversible focal infarction, whereas the demyelinating agent LPC induces diffused reversible WM injury. A limitation of ET-1 is that it also has receptor-mediated effects on OPC and astrocytes. Therefore, use of ET-1 as an agent to induce ischemic stroke in WM may confound the interpretation of neural repair tissue outcomes because these outcomes could be due to the direct ischemic effect of ET-1, or due to the effect of ET-1 on astrocytes and OPCs. Third, the dose of these agents at one single location or multiple locations of the same WM region should also be considered for a sustained WM injury model. Because the effectiveness of ET-1 in mice is still questionable, a combination of ET-1 and L-NIO can also be considered as an option for the stroke-related WM injury model. These are some of the basic considerations to optimize chemical-induced WM injury models. Some considerations for other WM injury models that are beyond the scope of this review can be found in these articles (11–16, 19, 20).

## Conclusion

Given the advances in various animal models of WM injury developed to understand the mechanisms of disease, a focus on disease management continues to increase. The crucial need to understand the cellular and molecular events that accompany the progressive phases of WM injury and demyelination in patients has led to the use of animal models. However, there has only been moderate success in translation using the proposed WM injury models, especially for mimicking functional deficits that occur in patients. Although current animal models do not reproduce all the clinical features of WM disease, they provide valuable information regarding disease mechanisms and disease-related neurobiological pathways, and they assist in testing possible interventions. The fact that the human brain differs considerably from rodents must be appreciated: such differences account for variations in lesion volumes and behavioral outcomes. In finding novel therapies for WM injuries, the first and foremost requirement is an in-depth knowledge of the neuropathology associated with the disorder while taking into account the complexity of WM and its distribution in the brain. Such an effort on development of optimized WM preclinical models holds great promise in leading to a comprehensive understanding of the disease mechanisms and in finding novel therapies that can alleviate the suffering of a significant portion of the population that is encountering this devastating disease.

## Author Contributions

AA, SD, and IS contributed to the design of the work, IS and AA participated in data acquisition, analysis, interpretation, and drafting the manuscript, and JF contributed to the data acquisition and analysis and drafting the manuscript. SN and SD contributed to the conception and design of the work and critically revised the manuscript. All authors agreed with final version of the manuscript, and agree to be accountable for all aspects of the work.

## Conflict of Interest Statement

The authors declare that the research was conducted in the absence of any commercial or financial relationships that could be construed as a potential conflict of interest.

## Abbreviations

CBF, cerebral blood flow; CC, corpus callosum; EB, ethidium bromide; ET-1, endothelin 1; eNOS, endothelial NOS; L-NIO, L-N^5^-(1-iminoethyl)ornithine; LPC, lysophosphatidylcholine; LPS, lipopolysaccharide; PLIC, posterior limb internal capsule; WM, white matter.

## References

[1] Zhang K Sejnowski TJ. A universal scaling law between gray matter and white matter of cerebral cortex. Proc Natl Acad Sci U S A (2000) 97:5621–6. 10.1073/pnas.090504197 10792049 PMC25878

[2] Leys D Englund E Del Ser T Inzitari D Fazekas F Bornstein N White matter changes in stroke patients. Relationship with stroke subtype and outcome. Eur Neurol (1999) 42:67–75. 10.1159/000069414 10473977

[3] Pantoni L Leys D Fazekas F Longstreth WT Jr Inzitari D Wallin A Role of white matter lesions in cognitive impairment of vascular origin. Alzheimer Dis Assoc Disord (1999) 13(Suppl 3):S49–54. 10.1097/00002093-199912001-00008 10609681

[4] Goldberg MP Ransom BR New light on white matter. Stroke (2003) 34:330–2. 10.1161/01.STR.0000054048.22626.B9 12574526

[5] Leviton A Gilles F Neff R Yaney P. Multivariate analysis of risk of perinatal telencephalic leucoencephalopathy. Am J Epidemiol (1976) 104:621–6. 998609 10.1093/oxfordjournals.aje.a112340

[6] Prins ND Van Dijk EJ Den Heijer T Vermeer SE Koudstaal PJ Oudkerk M Cerebral white matter lesions and the risk of dementia. Arch Neurol (2004) 61:1531–4. 10.1001/archneur.61.10.1531 15477506

[7] Kaur C Sivakumar V Ang LS Sundaresan A. Hypoxic damage to the periventricular white matter in neonatal brain: role of vascular endothelial growth factor, nitric oxide and excitotoxicity. J Neurochem (2006) 98:1200–16. 10.1111/j.1471-4159.2006.03964.x 16787408

[8] Puig J Pedraza S Blasco G Daunis IEJ Prados F Remollo S Acute damage to the posterior limb of the internal capsule on diffusion tensor tractography as an early imaging predictor of motor outcome after stroke. AJNR Am J Neuroradiol (2011) 32:857–63. 10.3174/ajnr.A2400 21474629 PMC7965569

[9] Hedna VS Jain S Rabbani O Nadeau SE. Mechanisms of arm paresis in middle cerebral artery distribution stroke: pilot study. J Rehabil Res Dev (2013) 50:1113–22. 10.1682/JRRD.2012.10.0194 24458897

[10] Shi H Hu X Leak RK Shi Y An C Suenaga J Demyelination as a rational therapeutic target for ischemic or traumatic brain injury. Exp Neurol (2015). 10.1016/j.expneurol.2015.03.017 PMC4581889 25819104

[11] Assareh A Mather KA Schofield PR Kwok JB Sachdev PS. The genetics of white matter lesions. CNS Neurosci Ther (2011) 17:525–40. 10.1111/j.1755-5949.2010.00181.x 21951372 PMC6493881

[12] Matute C Ransom BR. Roles of white matter in central nervous system pathophysiologies. ASN Neuro (2012) 4:e00079. 10.1042/AN20110060 22313331 PMC3310305

[13] Sozmen EG Hinman JD Carmichael ST. Models that matter: white matter stroke models. Neurotherapeutics (2012) 9:349–58. 10.1007/s13311-012-0106-0 22362423 PMC3337019

[14] Matute C Domercq M Perez-Samartin A Ransom BR Protecting white matter from stroke injury. Stroke (2013) 44:1204–11. 10.1161/STROKEAHA.112.658328 23212168

[15] Falcone GJ Malik R Dichgans M Rosand J. Current concepts and clinical applications of stroke genetics. Lancet Neurol (2014) 13:405–18. 10.1016/S1474-4422(14)70029-8 24646874

[16] Fern RF Matute C Stys PK. White matter injury: ischemic and nonischemic. Glia (2014) 62:1780–9. 10.1002/glia.22722 25043122

[17] Armstrong RC Mierzwa AJ Marion CM Sullivan GM. White matter involvement after TBI: clues to axon and myelin repair capacity. Exp Neurol (2015). 10.1016/j.expneurol.2015.02.011 25697845

[18] Glushakova OY Johnson D Hayes RL. Delayed increases in microvascular pathology after experimental traumatic brain injury are associated with prolonged inflammation, blood-brain barrier disruption, and progressive white matter damage. J Neurotrauma (2014) 31:1180–93. 10.1089/neu.2013.3080 24564198

[19] Weaver J Jalal FY Yang Y Thompson J Rosenberg GA Liu KJ. Tissue oxygen is reduced in white matter of spontaneously hypertensive-stroke prone rats: a longitudinal study with electron paramagnetic resonance. J Cereb Blood Flow Metab (2014) 34:890–6. 10.1038/jcbfm.2014.35 24549186 PMC4013771

[20] Jalal FY Yang Y Thompson JF Roitbak T Rosenberg GA. Hypoxia-induced neuroinflammatory white-matter injury reduced by minocycline in SHR/SP. J Cereb Blood Flow Metab (2015) 35(7):1145–53. 10.1038/jcbfm.2015.21 25712499 PMC4640265

[21] Sozmen EG Kolekar A Havton LA Carmichael ST. A white matter stroke model in the mouse: axonal damage, progenitor responses and MRI correlates. J Neurosci Methods (2009) 180:261–72. 10.1016/j.jneumeth.2009.03.017 19439360 PMC4697458

[22] Horie N Maag AL Hamilton SA Shichinohe H Bliss TM Steinberg GK. Mouse model of focal cerebral ischemia using endothelin-1. J Neurosci Methods (2008) 173:286–90. 10.1016/j.jneumeth.2008.06.013 18621079 PMC2572560

[23] Hughes PM Anthony DC Ruddin M Botham MS Rankine EL Sablone M Focal lesions in the rat central nervous system induced by endothelin-1. J Neuropathol Exp Neurol (2003) 62:1276–86. 14692703 10.1093/jnen/62.12.1276

[24] Frost SB Barbay S Mumert ML Stowe AM Nudo RJ. An animal model of capsular infarct: endothelin-1 injections in the rat. Behav Brain Res (2006) 169:206–11. 10.1016/j.bbr.2006.01.014 16497394

[25] Lecrux C McCabe C Weir CJ Gallagher L Mullin J Touzani O Effects of magnesium treatment in a model of internal capsule lesion in spontaneously hypertensive rats. Stroke (2008) 39:448–54. 10.1161/STROKEAHA.107.492934 18174487

[26] Blasi F Whalen MJ Ayata C. Lasting pure-motor deficits after focal posterior internal capsule white-matter infarcts in rats. J Cereb Blood Flow Metab (2015) 35:977–84. 10.1038/jcbfm.2015.7 25649992 PMC4640262

[27] He Z Yamawaki T Yang S Day AL Simpkins JW Naritomi H. Experimental model of small deep infarcts involving the hypothalamus in rats: changes in body temperature and postural reflex. Stroke (1999) 30:2743–51. 10.1161/01.STR.30.12.2743 10583006

[28] Tanaka Y Imai H Konno K Miyagishima T Kubota C Puentes S Experimental model of lacunar infarction in the gyrencephalic brain of the miniature pig: neurological assessment and histological, immunohistochemical, and physiological evaluation of dynamic corticospinal tract deformation. Stroke (2008) 39:205–12. 10.1161/STROKEAHA.107.489906 18048856

[29] Jean I Allamargot C Barthelaix-Pouplard A Fressinaud C. Axonal lesions and PDGF-enhanced remyelination in the rat corpus callosum after lysolecithin demyelination. Neuroreport (2002) 13:627–31. 10.1097/00001756-200204160-00018 11973459

[30] Jean I Lavialle C Barthelaix-Pouplard A Fressinaud C. Neurotrophin-3 specifically increases mature oligodendrocyte population and enhances remyelination after chemical demyelination of adult rat CNS. Brain Res (2003) 972:110–8. 10.1016/S0006-8993(03)02510-1 12711083

[31] Pham LD Hayakawa K Seo JH Nguyen MN Som AT Lee BJ Crosstalk between oligodendrocytes and cerebral endothelium contributes to vascular remodeling after white matter injury. Glia (2012) 60:875–81. 10.1002/glia.22320 22392631 PMC3325331

[32] Hinman JD Rasband MN Carmichael ST. Remodeling of the axon initial segment after focal cortical and white matter stroke. Stroke (2013) 44:182–9. 10.1161/STROKEAHA.112.668749 23233385 PMC3973016

[33] Clarner T Diederichs F Berger K Denecke B Gan L Van Der Valk P Myelin debris regulates inflammatory responses in an experimental demyelination animal model and multiple sclerosis lesions. Glia (2012) 60:1468–80. 10.1002/glia.22367 22689449

[34] Ni J Ohta H Matsumoto K Watanabe H. Progressive cognitive impairment following chronic cerebral hypoperfusion induced by permanent occlusion of bilateral carotid arteries in rats. Brain Res (1994) 653:231–6. 10.1016/0006-8993(94)90394-8 7982056

[35] Shibata M Ohtani R Ihara M Tomimoto H. White matter lesions and glial activation in a novel mouse model of chronic cerebral hypoperfusion. Stroke (2004) 35:2598–603. 10.1161/01.STR.0000143725.19053.60 15472111

[36] Shibata M Yamasaki N Miyakawa T Kalaria RN Fujita Y Ohtani R Selective impairment of working memory in a mouse model of chronic cerebral hypoperfusion. Stroke (2007) 38:2826–32. 10.1161/STROKEAHA.107.490151 17761909

[37] Hattori H Takeda M Kudo T Nishimura T Hashimoto S. Cumulative white matter changes in the gerbil brain under chronic cerebral hypoperfusion. Acta Neuropathol (1992) 84:437–42. 10.1007/BF00227672 1441925

[38] Kudo T Tada K Takeda M Nishimura T. Learning impairment and microtubule-associated protein 2 decrease in gerbils under chronic cerebral hypoperfusion. Stroke (1990) 21:1205–9. 10.1161/01.STR.21.8.1205 2389302

[39] Kudo T Takeda M Tanimukai S Nishimura T. Neuropathologic changes in the gerbil brain after chronic hypoperfusion. Stroke (1993) 24:259–64. 10.1161/01.STR.24.2.259 7678472

[40] Hattori Y Enmi J Kitamura A Yamamoto Y Saito S Takahashi Y A novel mouse model of subcortical infarcts with dementia. J Neurosci (2015) 35:3915–28. 10.1523/JNEUROSCI.3970-14.2015 25740520 PMC6605574

[41] Lubics A Reglodi D Tamas A Kiss P Szalai M Szalontay L Neurological reflexes and early motor behavior in rats subjected to neonatal hypoxic-ischemic injury. Behav Brain Res (2005) 157:157–65. 10.1016/j.bbr.2004.06.019 15617782

[42] Wang LS Zhou J Shao XM Tang XC. Huperzine A attenuates cognitive deficits and brain injury in neonatal rats after hypoxia-ischemia. Brain Res (2002) 949:162–70. 10.1016/S0006-8993(02)02977-3 12213312

[43] Shen Y Plane JM Deng W Mouse models of periventricular leukomalacia. J Vis Exp (2010) e1951 10.3791/1951 PMC3149994 20485263

[44] Rousset CI Kassem J Aubert A Planchenault D Gressens P Chalon S Maternal exposure to lipopolysaccharide leads to transient motor dysfunction in neonatal rats. Dev Neurosci (2013) 35:172–81. 10.1159/000346579 23445561

[45] Mathai S Booth LC Davidson JO Drury PP Fraser M Jensen EC Acute on chronic exposure to endotoxin in preterm fetal sheep. Am J Physiol Regul Integr Comp Physiol (2013) 304:R189–97. 10.1152/ajpregu.00388.2012 23235324

[46] Duncan JR Cock ML Scheerlinck JP Westcott KT McLean C Harding R White matter injury after repeated endotoxin exposure in the preterm ovine fetus. Pediatr Res (2002) 52:941–9. 10.1203/00006450-200212000-00021 12438674

[47] Ward P Counsell S Allsop J Cowan F Shen Y Edwards D Reduced fractional anisotropy on diffusion tensor magnetic resonance imaging after hypoxic-ischemic encephalopathy. Pediatrics (2006) 117:e619–30. 10.1542/peds.2005-0545 16510613

[48] Vannucci RC Vannucci SJ. Perinatal hypoxic-ischemic brain damage: evolution of an animal model. Dev Neurosci (2005) 27:81–6. 10.1159/000085978 16046840

[49] Rezaie P Dean A. Periventricular leukomalacia, inflammation and white matter lesions within the developing nervous system. Neuropathology (2002) 22:106–32. 10.1046/j.1440-1789.2002.00438.x 12416551

[50] Khwaja O Volpe JJ. Pathogenesis of cerebral white matter injury of prematurity. Arch Dis Child Fetal Neonatal Ed (2008) 93:F153–61. 10.1136/adc.2006.108837 18296574 PMC2569152

[51] Vannucci RC Connor JR Mauger DT Palmer C Smith MB Towfighi J Rat model of perinatal hypoxic-ischemic brain damage. J Neurosci Res (1999) 55:158–63. 10.1002/(SICI)1097-4547(19990115)55:2<158::AID-JNR3>3.0.CO;2-1 9972818

[52] Bracco L Piccini C Moretti M Mascalchi M Sforza A Nacmias B Alzheimer’s disease: role of size and location of white matter changes in determining cognitive deficits. Dement Geriatr Cogn Disord (2005) 20:358–66. 10.1159/000088562 16192726

[53] Kim YS Lee KM Choi BH Sohn EH Lee AY. Relation between the clock drawing test (CDT) and structural changes of brain in dementia. Arch Gerontol Geriatr (2009) 48:218–21. 10.1016/j.archger.2008.01.010 18313775

[54] Tullberg M Hultin L Ekholm S Mansson JE Fredman P Wikkelso C. White matter changes in normal pressure hydrocephalus and Binswanger disease: specificity, predictive value and correlations to axonal degeneration and demyelination. Acta Neurol Scand (2002) 105:417–26. 10.1034/j.1600-0404.2002.01189.x 12027829

[55] Cho ZH Law M Chi JG Choi SH Park SY Kammen A An anatomic review of thalamolimbic fiber tractography: ultra-high resolution direct visualization of thalamolimbic fibers anterior thalamic radiation, superolateral and inferomedial medial forebrain bundles, and newly identified septum pellucidum tract. World Neurosurg (2013) 83:54–61. 10.1016/j.wneu.2013.08.022 23973452

[56] Yanagisawa M Kurihara H Kimura S Goto K Masaki T. A novel peptide vasoconstrictor, endothelin, is produced by vascular endothelium and modulates smooth muscle Ca2+ channels. J Hypertens Suppl (1988) 6:S188–91. 10.1097/00004872-198812040-00056 2853725

[57] Yanagisawa M Kurihara H Kimura S Tomobe Y Kobayashi M Mitsui Y A novel potent vasoconstrictor peptide produced by vascular endothelial cells. Nature (1988) 332:411–5. 10.1038/332411a0 2451132

[58] Rubanyi GM Polokoff MA Endothelins: molecular biology, biochemistry, pharmacology, physiology, and pathophysiology. Pharmacol Rev (1994) 46:325–415. 7831383

[59] Kedzierski RM Yanagisawa M. Endothelin system: the double-edged sword in health and disease. Annu Rev Pharmacol Toxicol (2001) 41:851–76. 10.1146/annurev.pharmtox.41.1.851 11264479

[60] Giaid A Gibson SJ Ibrahim BN Legon S Bloom SR Yanagisawa M Endothelin 1, an endothelium-derived peptide, is expressed in neurons of the human spinal cord and dorsal root ganglia. Proc Natl Acad Sci U S A (1989) 86:7634–8. 10.1073/pnas.86.19.7634 2678110 PMC298121

[61] Arai H Hori S Aramori I Ohkubo H Nakanishi S. Cloning and expression of a cDNA encoding an endothelin receptor. Nature (1990) 348:730–2. 10.1038/348730a0 2175396

[62] Ehrenreich H Anderson RW Fox CH Rieckmann P Hoffman GS Travis WD Endothelins, peptides with potent vasoactive properties, are produced by human macrophages. J Exp Med (1990) 172:1741–8. 10.1084/jem.172.6.1741 1701822 PMC2188743

[63] MacCumber MW Ross CA Snyder SH. Endothelin in brain: receptors, mitogenesis, and biosynthesis in glial cells. Proc Natl Acad Sci U S A (1990) 87:2359–63. 10.1073/pnas.87.6.2359 2156267 PMC53686

[64] Lee ME De La Monte SM Ng SC Bloch KD Quertermous T. Expression of the potent vasoconstrictor endothelin in the human central nervous system. J Clin Invest (1990) 86:141–7. 10.1172/JCI114677 2195059 PMC296701

[65] Yoshizawa T Shinmi O Giaid A Yanagisawa M Gibson SJ Kimura S Endothelin: a novel peptide in the posterior pituitary system. Science (1990) 247:462–4. 10.1126/science.2405487 2405487

[66] Giaid A Gibson SJ Herrero MT Gentleman S Legon S Yanagisawa M Topographical localisation of endothelin mRNA and peptide immunoreactivity in neurones of the human brain. Histochemistry (1991) 95:303–14. 10.1007/BF00266781 2050550

[67] Koseki C Imai M Hirata Y Yanagisawa M Masaki T. Autoradiographic localization of [125I]-endothelin-1 binding sites in rat brain. Neurosci Res (1989) 6:581–5. 10.1016/0168-0102(89)90047-3 2677844

[68] Kadel KA Heistad DD Faraci FM. Effects of endothelin on blood vessels of the brain and choroid plexus. Brain Res (1990) 518:78–82. 10.1016/0006-8993(90)90956-C 2202492

[69] Sakurai T Yanagisawa M Masaki T. Molecular characterization of endothelin receptors. Trends Pharmacol Sci (1992) 13:103–8. 10.1016/0165-6147(92)90038-8 1315462

[70] Kuwaki T Kurihara H Cao WH Kurihara Y Unekawa M Yazaki Y Physiological role of brain endothelin in the central autonomic control: from neuron to knockout mouse. Prog Neurobiol (1997) 51:545–79. 10.1016/S0301-0082(96)00063-9 9153073

[71] Souza-Rodrigues RD Costa AM Lima RR Dos Santos CD Picanco-Diniz CW Gomes-Leal W. Inflammatory response and white matter damage after microinjections of endothelin-1 into the rat striatum. Brain Res (2008) 1200:78–88. 10.1016/j.brainres.2007.11.025 18289508

[72] Robinson MJ Macrae IM Todd M Reid JL McCulloch J. Reduction of local cerebral blood flow to pathological levels by endothelin-1 applied to the middle cerebral artery in the rat. Neurosci Lett (1990) 118:269–72. 10.1016/0304-3940(90)90644-O 2274283

[73] Willette RN Sauermelch CF. Abluminal effects of endothelin in cerebral microvasculature assessed by laser-Doppler flowmetry. Am J Physiol (1990) 259:H1688–93. 2124424 10.1152/ajpheart.1990.259.6.H1688

[74] Fuxe K Kurosawa N Cintra A Hallstrom A Goiny M Rosen L Involvement of local ischemia in endothelin-1 induced lesions of the neostriatum of the anaesthetized rat. Exp Brain Res (1992) 88:131–9. 10.1007/BF02259134 1541348

[75] Windle V Szymanska A Granter-Button S White C Buist R Peeling J An analysis of four different methods of producing focal cerebral ischemia with endothelin-1 in the rat. Exp Neurol (2006) 201:324–34. 10.1016/j.expneurol.2006.04.012 16740259

[76] Ziv I Fleminger G Djaldetti R Achiron A Melamed E Sokolovsky M. Increased plasma endothelin-1 in acute ischemic stroke. Stroke (1992) 23:1014–6. 10.1161/01.STR.23.7.1014 1615534

[77] Fuxe K Cintra A Andbjer B Anggard E Goldstein M Agnati LF Centrally administered endothelin-1 produces lesions in the brain of the male rat. Acta Physiol Scand (1989) 137:155–6. 10.1111/j.1748-1716.1989.tb08734.x 2678898

[78] Gilmour G Iversen SD O’neill MF Bannerman DM. The effects of intracortical endothelin-1 injections on skilled forelimb use: implications for modelling recovery of function after stroke. Behav Brain Res (2004) 150:171–83. 10.1016/j.bbr.2003.07.006 15033290

[79] Wang Y Jin K Greenberg DA. Neurogenesis associated with endothelin-induced cortical infarction in the mouse. Brain Res (2007) 1167:118–22. 10.1016/j.brainres.2007.06.065 17669376 PMC2098871

[80] Wiley KE Davenport AP. Endothelin receptor pharmacology and function in the mouse: comparison with rat and man. J Cardiovasc Pharmacol (2004) 44(Suppl 1):S4–6. 10.1097/01.fjc.0000166204.89426.20 15838332

[81] Babu BR Griffith OW. Design of isoform-selective inhibitors of nitric oxide synthase. Curr Opin Chem Biol (1998) 2:491–500. 10.1016/S1367-5931(98)80125-7 9736922

[82] Huang Z Huang PL Panahian N Dalkara T Fishman MC Moskowitz MA. Effects of cerebral ischemia in mice deficient in neuronal nitric oxide synthase. Science (1994) 265:1883–5. 10.1126/science.7522345 7522345

[83] Zhao X Haensel C Araki E Ross ME Iadecola C. Gene-dosing effect and persistence of reduction in ischemic brain injury in mice lacking inducible nitric oxide synthase. Brain Res (2000) 872:215–8. 10.1016/S0006-8993(00)02459-8 10924696

[84] Gross SS Wolin MS Nitric oxide: pathophysiological mechanisms. Annu Rev Physiol (1995) 57:737–69. 10.1146/annurev.ph.57.030195.003513 7539995

[85] Scorziello A Santillo M Adornetto A Dell’aversano C Sirabella R Damiano S NO-induced neuroprotection in ischemic preconditioning stimulates mitochondrial Mn-SOD activity and expression via Ras/ERK1/2 pathway. J Neurochem (2007) 103:1472–80. 10.1111/j.1471-4159.2007.04845.x 17680990

[86] Chen ZY Wang L Asavaritkrai P Noguchi CT. Up-regulation of erythropoietin receptor by nitric oxide mediates hypoxia preconditioning. J Neurosci Res (2010) 88:3180–8. 10.1002/jnr.22473 20806411

[87] Peng B Guo QL He ZJ Ye Z Yuan YJ Wang N Remote ischemic postconditioning protects the brain from global cerebral ischemia/reperfusion injury by up-regulating endothelial nitric oxide synthase through the PI3K/Akt pathway. Brain Res (2012) 1445:92–102. 10.1016/j.brainres.2012.01.033 22325092

[88] Widdop RE Gardiner SM Kemp PA Bennett T. The influence of atropine and atenolol on the cardiac haemodynamic effects of NG-nitro-L-arginine methyl ester in conscious, Long Evans rats. Br J Pharmacol (1992) 105:653–6. 10.1111/j.1476-5381.1992.tb09034.x 1628153 PMC1908447

[89] Ferreira-Melo SE Yugar-Toledo JC Coelho OR De Luca IM Tanus-Santos JE Hyslop S Sildenafil reduces cardiovascular remodeling associated with hypertensive cardiomyopathy in NOS inhibitor-treated rats. Eur J Pharmacol (2006) 542:141–7. 10.1016/j.ejphar.2006.04.039 16806160

[90] Galasso JM Bazzett TJ Becker JB Albin RL. Synergistic effect of intrastriatal co-administration of L-NAME and quinolinic acid. Neuroreport (1995) 6:1505–8. 10.1097/00001756-199507310-00010 7579135

[91] Ashwal S Cole DJ Osborne S Osborne TN Pearce WJ. L-NAME reduces infarct volume in a filament model of transient middle cerebral artery occlusion in the rat pup. Pediatr Res (1995) 38:652–6. 10.1203/00006450-199511000-00004 8552429

[92] Quast MJ Wei J Huang NC. Nitric oxide synthase inhibitor NG-nitro-L-arginine methyl ester decreases ischemic damage in reversible focal cerebral ischemia in hyperglycemic rats. Brain Res (1995) 677:204–12. 10.1016/0006-8993(95)00134-C 7552244

[93] Ding-Zhou L Marchand-Verrecchia C Croci N Plotkine M Margaill I. L-NAME reduces infarction, neurological deficit and blood-brain barrier disruption following cerebral ischemia in mice. Eur J Pharmacol (2002) 457:137–46. 10.1016/S0014-2999(02)02686-9 12464359

[94] Mulligan MS Moncada S Ward PA. Protective effects of inhibitors of nitric oxide synthase in immune complex-induced vasculitis. Br J Pharmacol (1992) 107:1159–62. 10.1111/j.1476-5381.1992.tb13423.x 1281719 PMC1907958

[95] McCall TB Feelisch M Palmer RM Moncada S. Identification of N-iminoethyl-L-ornithine as an irreversible inhibitor of nitric oxide synthase in phagocytic cells. Br J Pharmacol (1991) 102:234–8. 10.1111/j.1476-5381.1991.tb12159.x 1710525 PMC1917886

[96] Blakemore WF. Ethidium bromide induced demyelination in the spinal cord of the cat. Neuropathol Appl Neurobiol (1982) 8:365–75. 10.1111/j.1365-2990.1982.tb00305.x 7177337

[97] Loy DN Magnuson DS Zhang YP Onifer SM Mills MD Cao QL Functional redundancy of ventral spinal locomotor pathways. J Neurosci (2002) 22:315–23. 11756515 10.1523/JNEUROSCI.22-01-00315.2002 PMC6757623

[98] Loy DN Talbott JF Onifer SM Mills MD Burke DA Dennison JB Both dorsal and ventral spinal cord pathways contribute to overground locomotion in the adult rat. Exp Neurol (2002) 177:575–80. 10.1006/exnr.2002.7959 12429203

[99] Kuypers NJ James KT Enzmann GU Magnuson DS Whittemore SR. Functional consequences of ethidium bromide demyelination of the mouse ventral spinal cord. Exp Neurol (2013) 247:615–22. 10.1016/j.expneurol.2013.02.014 23466931 PMC3742572

[100] Woodruff RH Franklin RJ. Demyelination and remyelination of the caudal cerebellar peduncle of adult rats following stereotaxic injections of lysolecithin, ethidium bromide, and complement/anti-galactocerebroside: a comparative study. Glia (1999) 25:216–28. 10.1002/(SICI)1098-1136(19990201)25:3<216::AID-GLIA2>3.0.CO;2-L 9932868

[101] Hayashi J Tanaka M Sato W Ozawa T Yonekawa H Kagawa Y Effects of ethidium bromide treatment of mouse cells on expression and assembly of nuclear-coded subunits of complexes involved in the oxidative phosphorylation. Biochem Biophys Res Commun (1990) 167:216–21. 10.1016/0006-291X(90)91753-F 2310389

[102] Hayakawa T Noda M Yasuda K Yorifuji H Taniguchi S Miwa I Ethidium bromide-induced inhibition of mitochondrial gene transcription suppresses glucose-stimulated insulin release in the mouse pancreatic beta-cell line betaHC9. J Biol Chem (1998) 273:20300–7. 10.1074/jbc.273.32.20300 9685380

[103] Blakemore WF. The case for a central nervous system (CNS) origin for the Schwann cells that remyelinate CNS axons following concurrent loss of oligodendrocytes and astrocytes. Neuropathol Appl Neurobiol (2005) 31:1–10. 10.1111/j.1365-2990.2005.00637.x 15634226

[104] Titsworth WL Onifer SM Liu NK Xu XM. Focal phospholipases A2 group III injections induce cervical white matter injury and functional deficits with delayed recovery concomitant with Schwann cell remyelination. Exp Neurol (2007) 207:150–62. 10.1016/j.expneurol.2007.06.010 17678647

[105] Matsumoto T Kobayashi T Kamata K. Role of lysophosphatidylcholine (LPC) in atherosclerosis. Curr Med Chem (2007) 14:3209–20. 10.2174/092986707782793899 18220755

[106] Larsen PH Wells JE Stallcup WB Opdenakker G Yong VW. Matrix metalloproteinase-9 facilitates remyelination in part by processing the inhibitory NG2 proteoglycan. J Neurosci (2003) 23:11127–35. 14657171 10.1523/JNEUROSCI.23-35-11127.2003 PMC6741053

[107] Kotter MR Zhao C Van Rooijen N Franklin RJ. Macrophage-depletion induced impairment of experimental CNS remyelination is associated with a reduced oligodendrocyte progenitor cell response and altered growth factor expression. Neurobiol Dis (2005) 18:166–75. 10.1016/j.nbd.2004.09.019 15649707

[108] Birgbauer E Rao TS Webb M. Lysolecithin induces demyelination in vitro in a cerebellar slice culture system. J Neurosci Res (2004) 78:157–66. 10.1002/jnr.20248 15378614

[109] Ousman SS David S. Lysophosphatidylcholine induces rapid recruitment and activation of macrophages in the adult mouse spinal cord. Glia (2000) 30:92–104. 10.1002/(SICI)1098-1136(200003)30:1<92::AID-GLIA10>3.3.CO;2-N 10696148

[110] Ousman SS David S. MIP-1alpha, MCP-1, GM-CSF, and TNF-alpha control the immune cell response that mediates rapid phagocytosis of myelin from the adult mouse spinal cord. J Neurosci (2001) 21:4649–56. 11425892 10.1523/JNEUROSCI.21-13-04649.2001 PMC6762369

[111] Ghasemlou N Jeong SY Lacroix S David S. T cells contribute to lysophosphatidylcholine-induced macrophage activation and demyelination in the CNS. Glia (2007) 55:294–302. 10.1002/glia.20449 17096403

[112] Jeffery ND Blakemore WF. Remyelination of mouse spinal cord axons demyelinated by local injection of lysolecithin. J Neurocytol (1995) 24:775–81. 10.1007/BF01191213 8586997

[113] Yoon BH Jun JK Romero R Park KH Gomez R Choi JH Amniotic fluid inflammatory cytokines (interleukin-6, interleukin-1beta, and tumor necrosis factor-alpha), neonatal brain white matter lesions, and cerebral palsy. Am J Obstet Gynecol (1997) 177:19–26. 10.1016/S0002-9378(97)70276-X 9240577

[114] Sheldon RA Chuai J Ferriero DM. A rat model for hypoxic-ischemic brain damage in very premature infants. Biol Neonate (1996) 69:327–41. 10.1159/000244327 8790911

[115] Vannucci RC Brucklacher RM Vannucci SJ. Glycolysis and perinatal hypoxic-ischemic brain damage. Dev Neurosci (2005) 27:185–90. 10.1159/000085991 16046853

[116] Back SA Han BH Luo NL Chricton CA Xanthoudakis S Tam J Selective vulnerability of late oligodendrocyte progenitors to hypoxia-ischemia. J Neurosci (2002) 22:455–63. 11784790 10.1523/JNEUROSCI.22-02-00455.2002 PMC6758669

[117] Yatomi Y Tanaka R Shimura H Miyamoto N Yamashiro K Takanashi M Chronic brain ischemia induces the expression of glial glutamate transporter EAAT2 in subcortical white matter. Neuroscience (2013) 244:113–21. 10.1016/j.neuroscience.2013.04.018 23602887

[118] Ueno M Tomimoto H Akiguchi I Wakita H Sakamoto H. Blood-brain barrier disruption in white matter lesions in a rat model of chronic cerebral hypoperfusion. J Cereb Blood Flow Metab (2002) 22:97–104. 10.1097/00004647-200201000-00012 11807399

[119] Goepfert AR Andrews WW Carlo W Ramsey PS Cliver SP Goldenberg RL Umbilical cord plasma interleukin-6 concentrations in preterm infants and risk of neonatal morbidity. Am J Obstet Gynecol (2004) 191:1375–81. 10.1016/j.ajog.2004.06.086 15507968

[120] Cai Z Pan ZL Pang Y Evans OB Rhodes PG. Cytokine induction in fetal rat brains and brain injury in neonatal rats after maternal lipopolysaccharide administration. Pediatr Res (2000) 47:64–72. 10.1203/00006450-200001000-00013 10625084

[121] Bell MJ Hallenbeck JM Gallo V. Determining the fetal inflammatory response in an experimental model of intrauterine inflammation in rats. Pediatr Res (2004) 56:541–6. 10.1203/01.PDR.0000139407.89883.6B 15295096

[122] Kohmura Y Kirikae T Kirikae F Nakano M Sato I. Lipopolysaccharide (LPS)-induced intra-uterine fetal death (IUFD) in mice is principally due to maternal cause but not fetal sensitivity to LPS. Microbiol Immunol (2000) 44:897–904. 10.1111/j.1348-0421.2000.tb02581.x 11145270

[123] Paintlia MK Paintlia AS Khan M Singh I Singh AK. Modulation of peroxisome proliferator-activated receptor-alpha activity by N-acetyl cysteine attenuates inhibition of oligodendrocyte development in lipopolysaccharide stimulated mixed glial cultures. J Neurochem (2008) 105:956–70. 10.1111/j.1471-4159.2007.05199.x 18205750 PMC2659629

[124] Chamnanvanakij S Margraf LR Burns D Perlman JM. Apoptosis and white matter injury in preterm infants. Pediatr Dev Pathol (2002) 5:184–9. 10.1007/s10024-001-0205-0 11910514

[125] Yonezawa M Back SA Gan X Rosenberg PA Volpe JJ. Cystine deprivation induces oligodendroglial death: rescue by free radical scavengers and by a diffusible glial factor. J Neurochem (1996) 67:566–73. 10.1046/j.1471-4159.1996.67020566.x 8764581

[126] Baerwald KD Popko B. Developing and mature oligodendrocytes respond differently to the immune cytokine interferon-gamma. J Neurosci Res (1998) 52:230–9. 10.1002/(SICI)1097-4547(19980415)52:2<230::AID-JNR11>3.0.CO;2-B 9579413

[127] Sharkey J Ritchie IM Kelly PA. Perivascular microapplication of endothelin-1: a new model of focal cerebral ischaemia in the rat. J Cereb Blood Flow Metab (1993) 13:865–71. 10.1038/jcbfm.1993.108 8360292

[128] Hunter AJ Green AR Cross AJ. Animal models of acute ischaemic stroke: can they predict clinically successful neuroprotective drugs? Trends Pharmacol Sci (1995) 16:123–8. 10.1016/S0165-6147(00)88999-3 7610497

[129] Leviton A Gilles FH An epidemiologic study of perinatal telencephalic leucoencephalopathy in an autopsy population. J Neurol Sci (1973) 18:53–66. 10.1016/0022-510X(73)90020-8 4690638

[130] Leviton A Gilles FH. Are hypertrophic astrocytes a sufficient criterion of perinatal telencephalic leucoencephalopathy? J Neurol Neurosurg Psychiatry (1973) 36:383–8. 10.1136/jnnp.36.3.383 4714100 PMC494337

[131] Gilles FH Leviton A Kerr CS. Endotoxin leucoencephalopathy in the telencephalon of the newborn kitten. J Neurol Sci (1976) 27:183–91. 10.1016/0022-510X(76)90060-5 1249585

[132] Leviton A Gilles FH. Acquired perinatal leukoencephalopathy. Ann Neurol (1984) 16:1–8. 10.1002/ana.410160102 6465860

[133] Deng Y Xie D Fang M Zhu G Chen C Zeng H Astrocyte-derived proinflammatory cytokines induce hypomyelination in the periventricular white matter in the hypoxic neonatal brain. PLoS One (2014) 9:e87420. 10.1371/journal.pone.0087420 24498101 PMC3909103

[134] Mifsud G Zammit C Muscat R Di Giovanni G Valentino M. Oligodendrocyte pathophysiology and treatment strategies in cerebral ischemia. CNS Neurosci Ther (2014) 20:603–12. 10.1111/cns.12263 24703424 PMC6493108

[135] Stys PK Ransom BR Waxman SG Davis PK. Role of extracellular calcium in anoxic injury of mammalian central white matter. Proc Natl Acad Sci U S A (1990) 87:4212–6. 10.1073/pnas.87.11.4212 2349231 PMC54078

[136] Stys PK Waxman SG Ransom BR. Ionic mechanisms of anoxic injury in mammalian CNS white matter: role of Na+ channels and Na(+)-Ca2+ exchanger. J Neurosci (1992) 12:430–9. 1311030 10.1523/JNEUROSCI.12-02-00430.1992 PMC6575619

[137] Baltan S. Ischemic injury to white matter: an age-dependent process. Neuroscientist (2009) 15:126–33. 10.1177/1073858408324788 19307420

[138] Ouardouz M Nikolaeva MA Coderre E Zamponi GW McRory JE Trapp BD Depolarization-induced Ca2+ release in ischemic spinal cord white matter involves L-type Ca2+ channel activation of ryanodine receptors. Neuron (2003) 40:53–63. 10.1016/j.neuron.2003.08.016 14527433

[139] Agrawal SK Fehlings MG. Role of NMDA and non-NMDA ionotropic glutamate receptors in traumatic spinal cord axonal injury. J Neurosci (1997) 17:1055–63. 8994060 10.1523/JNEUROSCI.17-03-01055.1997 PMC6573164

[140] Matute C Alberdi E Domercq M Sanchez-Gomez MV Perez-Samartin A Rodriguez-Antiguedad A Excitotoxic damage to white matter. J Anat (2007) 210:693–702. 10.1111/j.1469-7580.2007.00733.x 17504270 PMC2375761

[141] Kadhim H Tabarki B Verellen G De Prez C Rona AM Sebire G. Inflammatory cytokines in the pathogenesis of periventricular leukomalacia. Neurology (2001) 56:1278–84. 10.1212/WNL.56.10.1278 11376173

[142] Kadhim H Tabarki B De Prez C Rona AM Sebire G. Interleukin-2 in the pathogenesis of perinatal white matter damage. Neurology (2002) 58:1125–8. 10.1212/WNL.58.7.1125 11940709

[143] Folkerth RD Keefe RJ Haynes RL Trachtenberg FL Volpe JJ Kinney HC. Interferon-gamma expression in periventricular leukomalacia in the human brain. Brain Pathol (2004) 14:265–74. 10.1111/j.1750-3639.2004.tb00063.x 15446581 PMC8095901

[144] Dammann O Leviton A. Maternal intrauterine infection, cytokines, and brain damage in the preterm newborn. Pediatr Res (1997) 42:1–8. 10.1203/00006450-199707000-00001 9212029

[145] Smith SE Li J Garbett K Mirnics K Patterson PH. Maternal immune activation alters fetal brain development through interleukin-6. J Neurosci (2007) 27:10695–702. 10.1523/JNEUROSCI.2178-07.2007 17913903 PMC2387067

[146] Deguchi K Oguchi K Takashima S. Characteristic neuropathology of leukomalacia in extremely low birth weight infants. Pediatr Neurol (1997) 16:296–300. 10.1016/S0887-8994(97)00041-6 9258961

[147] Meng SZ Arai Y Deguchi K Takashima S. Early detection of axonal and neuronal lesions in prenatal-onset periventricular leukomalacia. Brain Dev (1997) 19:480–4. 10.1016/S0387-7604(97)00068-5 9408595

[148] Bakiri Y Hamilton NB Karadottir R Attwell D. Testing NMDA receptor block as a therapeutic strategy for reducing ischaemic damage to CNS white matter. Glia (2008) 56:233–40. 10.1002/glia.20608 18046734 PMC2863073

[149] Bakiri Y Burzomato V Frugier G Hamilton NB Karadottir R Attwell D. Glutamatergic signaling in the brain’s white matter. Neuroscience (2009) 158:266–74. 10.1016/j.neuroscience.2008.01.015 18314276

[150] Dewar D Underhill SM Goldberg MP. Oligodendrocytes and ischemic brain injury. J Cereb Blood Flow Metab (2003) 23:263–74. 10.1097/00004647-200303000-00001 12621301

[151] Yoshizaki K Adachi K Kataoka S Watanabe A Tabira T Takahashi K Chronic cerebral hypoperfusion induced by right unilateral common carotid artery occlusion causes delayed white matter lesions and cognitive impairment in adult mice. Exp Neurol (2008) 210:585–91. 10.1016/j.expneurol.2007.12.005 18222425

[152] Sotak CH. The role of diffusion tensor imaging in the evaluation of ischemic brain injury – a review. NMR Biomed (2002) 15:561–9. 10.1002/nbm.786 12489102

[153] Deng W. Neurobiology of injury to the developing brain. Nat Rev Neurol (2010) 6:328–36. 10.1038/nrneurol.2010.53 20479779

[154] Morken TS Wideroe M Vogt C Lydersen S Havnes M Skranes J Longitudinal diffusion tensor and manganese-enhanced MRI detect delayed cerebral gray and white matter injury after hypoxia-ischemia and hyperoxia. Pediatr Res (2013) 73:171–9. 10.1038/pr.2012.170 23174702

[155] Fuchtemeier M Brinckmann MP Foddis M Kunz A Po C Curato C Vascular change and opposing effects of the angiotensin type 2 receptor in a mouse model of vascular cognitive impairment. J Cereb Blood Flow Metab (2015) 35:476–84. 10.1038/jcbfm.2014.221 25492118 PMC4348389

[156] Van de Looij Y Chatagner A Quairiaux C Gruetter R Huppi PS Sizonenko SV. Multi-modal assessment of long-term erythropoietin treatment after neonatal hypoxic-ischemic injury in rat brain. PLoS One (2014) 9:e95643. 10.1371/journal.pone.0095643 24755676 PMC3995802

[157] Calabrese E Du F Garman RH Johnson GA Riccio C Tong LC Diffusion tensor imaging reveals white matter injury in a rat model of repetitive blast-induced traumatic brain injury. J Neurotrauma (2014) 31:938–50. 10.1089/neu.2013.3144 24392843 PMC4012630

[158] Donovan V Kim C Anugerah AK Coats JS Oyoyo U Pardo AC Repeated mild traumatic brain injury results in long-term white-matter disruption. J Cereb Blood Flow Metab (2014) 34:715–23. 10.1038/jcbfm.2014.6 24473478 PMC3982100

[159] Khan MB Hoda MN Vaibhav K Giri S Wang P Waller JL Remote ischemic postconditioning: harnessing endogenous protection in a murine model of vascular cognitive impairment. Transl Stroke Res (2015) 6:69–77. 10.1007/s12975-014-0374-6 25351177 PMC4297613

[160] Biran V Joly L-M Héron A Vernet A Véga C Mariani J Glial activation in white matter following ischemia in the neonatal P7 rat brain. Exp Neurol (2006) 199:103–12. 10.1016/j.expneurol.2006.01.037 16697370

[161] Cui X Chopp M Zacharek A Ning R Ding X Roberts C Endothelial nitric oxide synthase regulates white matter changes via the BDNF/TrkB pathway after stroke in mice. PLoS One (2013) 8:e80358. 10.1371/journal.pone.0080358 24236179 PMC3827451

[162] Xiong M Li J Ma S-M Yang Y Zhou W-H. Effects of hypothermia on oligodendrocyte precursor cell proliferation, differentiation and maturation following hypoxia ischemia in vivo and in vitro. Exp Neurol (2013) 247:720–9. 10.1016/j.expneurol.2013.03.015 23524193

[163] Deng Y-P Sun Y Hu L Li Z-H Xu Q-M Pei Y-L Chondroitin sulfate proteoglycans impede myelination by oligodendrocytes after perinatal white matter injury. Exp Neurol (2015) 269:213–23. 10.1016/j.expneurol.2015.03.026 25862289

[164] Wenzelburger R Kopper F Frenzel A Stolze H Klebe S Brossmann A Hand coordination following capsular stroke. Brain (2005) 128:64–74. 10.1093/brain/awh317 15471902

[165] Schulz R Park C-H Boudrias M-H Gerloff C Hummel FC Ward NS. Assessing the integrity of corticospinal pathways from primary and secondary cortical motor areas after stroke. Stroke (2012) 43:2248–51. 10.1161/strokeaha.112.662619 22764214 PMC3477824

[166] Petoe MA Byblow WD De Vries EJM Krishnamurthy V Zhong CS Barber PA A template-based procedure for determining white matter integrity in the internal capsule early after stroke. Neuroimage Clin (2014) 4:695–700. 10.1016/j.nicl.2013.12.006 24936407 PMC4053651

[167] Song J Nair VA Young BM Walton LM Nigogosyan Z Remsik AB DTI measures track and predict motor function outcomes in stroke rehabilitation utilizing BCI technology. Front Hum Neurosci (2015) 9:195. 10.3389/fnhum.2015.00195 25964753 PMC4410488

